# Functionalization of Gadolinium Chelates Silica Nanoparticle through Silane Chemistry for Simultaneous MRI/^64^Cu PET Imaging

**DOI:** 10.1155/2018/7938267

**Published:** 2018-11-01

**Authors:** Vu-Long Tran, Vivek Thakare, Marco Natuzzi, Mathieu Moreau, Alexandra Oudot, Jean-Marc Vrigneaud, Alan Courteau, Cédric Louis, Stéphane Roux, Frédéric Boschetti, Franck Denat, Olivier Tillement, François Lux

**Affiliations:** ^1^Univ Lyon, Université Claude Bernard Lyon 1, CNRS, Institut Lumière Matière, F-69622 Lyon, France; ^2^Nano-H S.A.S., 2 Place de l'Europe, 38070 Saint Quentin Fallavier, France; ^3^CheMatech, 2 rue Pauline Kergomard, Dijon, France; ^4^Institut de Chimie Moléculaire de l'Université de Bourgogne (ICMUB), UMR CNRS 6302, Université de Bourgogne Franche-Comté, 9 Avenue Alain Savary, 21078 Dijon, France; ^5^Plateforme d'Imagerie et Radiothérapie Précliniques, Médecine Nucléaire, Georges-Francois LECLERC Cancer Center-UNICANCER, 21000 Dijon, France; ^6^Institut UTINAM, Université de Bourgogne Franche-Comté, CNRS UMR 6213, 16 Route de Gray, 25030 Besançon, France; ^7^NH TherAguix SAS, F69100 Villeurbanne, France

## Abstract

Multimodal nanoprobes are highly demanded for biomedical imaging applications to enhance the reliability of the diagnostic results. Among different types of nano-objects, ultrasmall silica gadolinium nanoparticle (SiGdNP) appears as a safe, effective, and versatile platform for this purpose. In this study, a new method to functionalize SiGdNP based on silane chemistry has been reported. Two types of chelating silanes (APTES-DOTAGA and APTES-NODAGA) have been synthesized and grafted on SiGdNP by a simple one-step protocol. This functionalization strategy requires no other reactants or catalyzers and does not compromise the ultrasmall size of the particles. NODAGA-functionalized particle has been labeled with ^64^Cu isotope and injected intravenously to mice bearing TS/A carcinoma tumor for biodistribution study to demonstrate its potential as a bimodal MRI/PET imaging agent. A fully integrated MRI/PET system was used to simultaneously monitor the distribution of the particle. The results showed that the functionalized particle maintained properties of a renal clearable NP which could rapidly escape through kidneys and had low retention in other organs, especially liver, even though its accumulation in the tumor was modest.

## 1. Introduction

**Figure 1:**
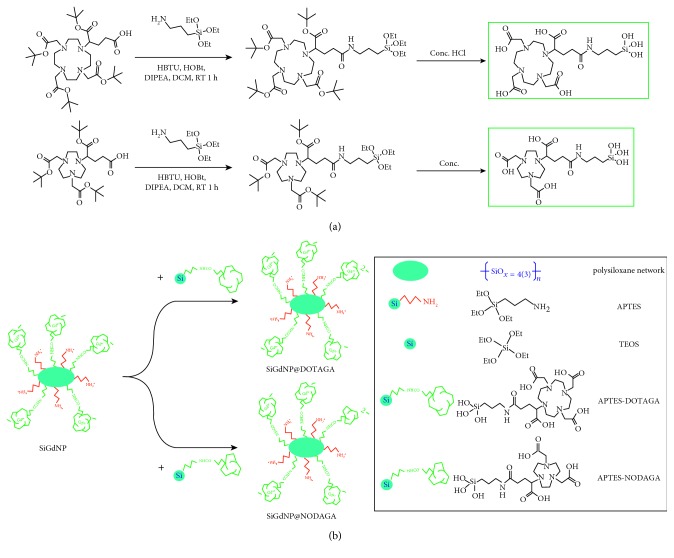
(a) The reaction scheme of the synthesis of APTES-DOTAGA (upper) and APTES-NODAGA (lower); (b) the reaction scheme of the functionalization of APTES-DOTAGA and APTES-NODAG on SiGdNP.

Biomedical imaging is composed of different sophisticated techniques ranging from ultrasonography (US), X-ray computed tomography (CT), magnetic resonance imaging (MRI), nuclear imaging, and optical imaging (OI). However, each of them has different drawbacks alongside with advantages. Among them, MRI is gaining more and more popularity in hospitals for precise imaging thanks to high spatial resolution and nonionizing nature of the radiation. Nevertheless, MRI is plagued by its low sensitivity and therefore, not an ideal technique for quantification of contrast agents. On the other hand, nuclear imaging, more specifically, single photon emission computed tomography (SPECT) or positron emission tomography (PET), offers precise quantification without being limited by penetrability issue as OI. Hence, more and more researches have been dedicated to the combination of MRI contrast agents and radioisotopes in one single object to correlate the images obtained by MRI and the ones acquired by nuclear imaging [1]. In this context, a bimodal probe which allows the localization of disease sites by both techniques should be highly desirable. Our group has developed an ultrasmall silica gadolinium nanoparticle (SiGdNP) as a probe for multimodal imaging and radiosensitization [2]. It is made of a polysiloxane core and displays several macrocyclic chelators DOTA (1,4,7,10-tetraazacyclododecane-1,4,7,10- tetraacetic acid) on the surface. Most of them complexed gadolinium ions (Gd^3+^) and acted as positive contrast agent for MRI. This type of nanoparticle (NP) was made from biocompatible materials. Moreover, it displays an ultrasmall hydrodynamic diameter (<5 nm) and is covered by both positively (amines) and negatively charged (DOTA chelates of Gd) groups. The latter helps distribute the opposite charges evenly and further minimize protein adsorption [3]. These parameters allow it to be quickly eliminated through kidneys and prevent the deposit of chelates in organs like liver, spleen, lung, or bone marrow. This characteristic combined with the high affinity of DOTA for gadolinium helps in avoiding possible release of toxic Gd ions [4–6]. Meanwhile, different radioisotopes, e.g., ^111^In, ^68^Ga, and ^89^Zr, used for nuclear imaging can be complexed to free chelators that existed from the beginning or being grafted postsynthetically [7–9]. Common methods for grafting free chelators on SiGdNP were to use NHS esters [8, 10] or isothiocyanate derivatives [9] of chelators to react with amine groups on the surface of the particles [9]. The materials required for these strategies have been well developed and commercialized. Moreover, these reactions are quite fast, straightforward, and well known. Nevertheless, there are still some limitations for these strategies. First of all, NHS esters and isothiocyanates are prone to be deactivated by hydrolysis. Hence, they cannot be stored for a long period of time even at low temperature in the desiccated condition. Second, these activated species can cross-react with amines present in targeting ligands that we want to functionalize, for example, *ε*-amine of lysine of a peptide. This implies complicated protecting/unprotecting strategies such as using tert-butyloxycarbonyl (Boc) or 9H-fluoren-9-ylmethoxycarbonyl (Fmoc) protecting groups [11]. In this study, a new strategy based on silane chemistry was proposed. The macrocyclic chelators, i.e., DOTAGA (1,4,7,10-tetraazacyclododecane-1-glutaric acid-4,7,10-triacetic acid) and NODAGA (1,4,7-triazacyclononane, 1-glutaric acid-4,7-diacetic acid) were coupled with APTES (aminopropyl triethoxysilane) (Figure 1(a)). These species were then used to functionalize SiGdNP by reacting specifically with available silanol groups, Si-OH, on the surface of the particle in a single step without the presence of any other reactants (Figure 1(b)). This strategy has been used by Ciccione et al. to successfully graft precisely certain ratios of fluorophores and different targeting peptides on a 80 nm silica nanoparticle to actively increase the internalization of particles in cancer cells [12]. The activities of silanes are not affected by hydrolysis. Hence, it can be stored and used for a long period of time. In addition, the unreacted silanes are highly soluble so that they should be removed easily by filtration. The functionalized particles were characterized with different analytical methods to prove the presence of free chelators on the particles. By increasing the quantity of silane precursors, the amount of free chelators can be increased proportionally. Finally, a simultaneous bimodal imaging experiment using a fully integrated MRI/PET system was performed on murine tumor model to demonstrate the potential of this type of NP. Radioisotope of copper, ^64^Cu, was chosen as positron emitter for PET modality. This isotope has a reasonable half-life (12.7 h) for pharmacokinetic study of SiGdNP which has a 1 h half-life in patients' blood (phase I trial NANO-RAD; ClinicalTrials.gov Identifier: NCT02820454). In addition, the energy of positron emission from ^64^Cu is relatively low (656 keV), and the lowest-energy positrons from a commonly used radiometal. This allows obtaining images with resolution as high as the ones obtained with clinically prevalent ^18^F isotope (640 keV) [13]. Regarding the choice of chelator, although the thermodynamic stability constant for Cu complex of NOTA (original structure of NODAGA) is comparable to the one of DOTA (logK_NOTA_ = 21.6 vs. logK_DOTA_ = 22.2), [14] different studies have shown higher *in vivo* stability of Cu complexes of NOTA, NODAGA, and their derivatives compared to the ones of DOTAGA and their derivatives [15–18]. Therefore, NODAGA functionalized particle was chosen to be radiolabeled with ^64^Cu for the biodistribution study.

## 2. Results and Discussion

### 2.1. Functionalization of NPs through Silane Chemistry

Two types of chelating silanes, i.e., APTES-DOTAGA and APTES-NODAGA, were synthesized from the reaction between APTES and either t-butyl protected DOTAGA ((*t*-Bu)_4_DOTAGA) or t-butyl protected NODAGA ((*t*-Bu)_3_NODAGA) (Figure 1(a)). The peptide bond was formed by using HBTU (2-(1*H*-benzotriazol-1-yl)-1,1,3,3-tetramethyluronium hexafluorophosphate) as coupling agent. After purification, APTES-(*t*-Bu)_4_DOTAGA and APTES-(*t*-Bu)_3_NODAGA, were deprotected by concentrated hydrochloric acid to obtain final products. The excess of acid was removed by evaporation. Solutions were lyophilized for storage.

**Figure 2:**
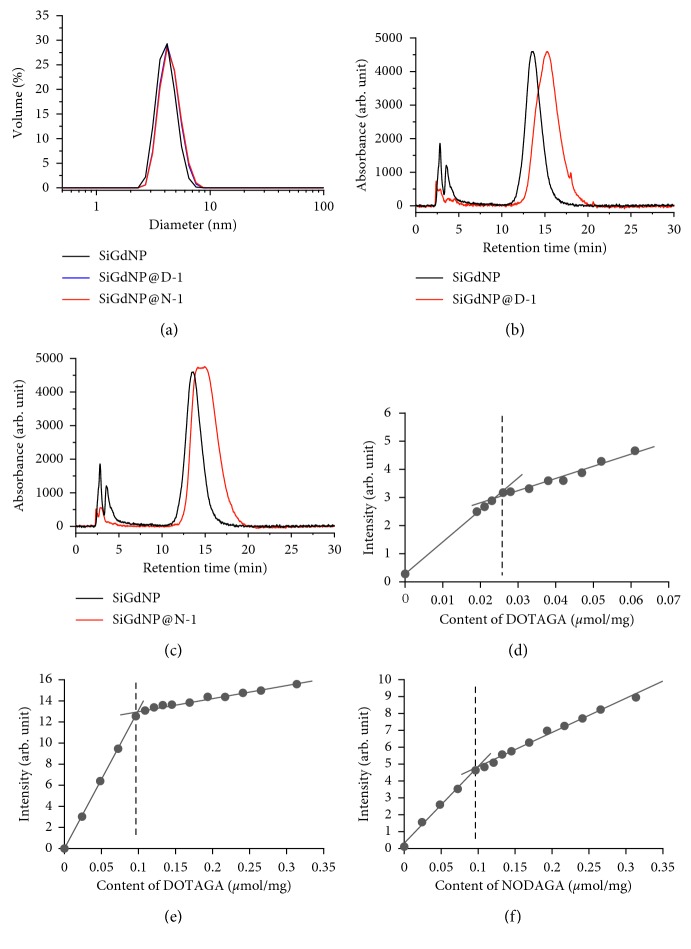
Physicochemical characterization during the functionalization of GdNP. (a) DLS diagrams of SiGdNP (black), SiGdNP @ D-1 (DOTAGA) (blue), and SiGdNP @ N-1 (NODAGA) (red). (b) HPLC chromatograms of SiGdNP (before functionalization) (black) and SiGdNP @ D-1 (after functionalization with DOTAGA at 80°C) (red). (c) HPLC chromatograms of SiGdNP (before functionalization) (black) and SiGdNP @ N-1 (after functionalization with NODAGA at 80°C) (red). The concentration of samples analyzed by HPLC was at 5 mM in Gd. Eu titration curves *λ*_ex_ = 395 nm, *λ*_em_ = 594 nm of SiGdNP (d), SiGdNP @ D-1 (e), and SiGdNP @ N-1 (f).

Then, each type of silanes was mixed with SiGdNP in molar ratio Gd : chelator = 10 : 1.5. Due to the steric hindrance of the macrocyclic chelators, the silanes, i.e., APTES-DOTAGA and APTES-NODAGA, are not likely to form stable particles. However, when being mixed with already-synthesized particles, they can form covalent siloxane bonds with free silanol groups or exchange with the already-existing silanized chelators on the surface of the particles. The remaining free silanes can be easily washed out by tangential filtration. The resulting NPs were referred to as SiGdNP @ D-1 (for DOTAGA functionalized particle) and SiGdNP @ N-1 (for NODAGA functionalized particle), respectively. Furthermore, lower temperature (40°C instead of 80°C) and lower total silanized chelator concentration including the chelators on the surface of the particles and added free chelators (ca. 58 mM instead of ca. 115 mM) were applied to see the effects on the homogeneity of the final NPs. These particles were referred to as SiGdNP @ D-2 and SiGdNP @ N-2. A series of samples with increasing amount of APTES-DOTAGA, i.e., Gd : chelator = 10 : 1.5, 10 : 2, and 10 : 4, was prepared to test the possibility of tailoring the amount of free chelators at the surface of the NP. These particles were referred to as SiGdNP @ D-2, 3, 4 accordingly. The experimental conditions of these particles were summarized in Table S1. Tables 1 and S2 show different characterization results of the NP before and after functionalization by chelating silanes at 80°C. *D*_H_ of the particles which only slight increase from 4.2 ± 0.8 nm for SiGdNP to 4.5 ± 0.9 nm for SiGdNP @ D-1 and SiGdNP @ N-1 indicated that the size was maintained ultrasmall (Figure 2(a)). On the other hand, other characteristics proved the presence of free chelators on the particles after the reaction. First of all, the zeta potentials at pH 7.0 decreased from +8.2 mV for SiGdNP to −8.3 mV for SiGdNP @ D-1 or −13.7 mV for SiGdNP @ N-1. Vibration band at 1730 cm^−1^ in IR spectra, corresponding to C=O stretching vibration band of free carboxylic acid, was detected (Figure S5). The retention time (*t*_R_) in chromatograms increased from 13.6 min for SiGdNP to 15.3 min for SiGdNP @ D-1 or 14.7 min for SiGdNP @ N-1 (Figures 2(b) and 2(c)). The content of free chelators quantified by Eu titration increased from 0.025 *µ*mol/mg for SiGdNP to 0.10 *µ*mol/mg for SiGdNP @ D-1 and SiGdNP @ N-1 (Figures 2(d)–2(f)). The principle of this titration method was described in details in a precedent paper. Briefly, a series of samples containing a fixed amount of NPs and an increasing amount of EuCl_3_ was prepared. Eu^3+^ has two specific phosphorescence emission peaks at 594 nm and 616 nm when being excited at 395 nm [19, 20].

When Eu^3+^ complexed with DOTAGA, it will be shielded from the quenching effect of O-H oscillator of water and emits much stronger luminescence signals. When the amount of Eu^3+^ increases, the intensity increases linearly until no DOTAGA is available for chelation. Then, the intensity will increase more slowly due only to the phosphorescence of free Eu^3+^ [8]. The content of Gd measured by ICP-OES decreased from 0.89 *µ*mol/mg for SiGdNP to 0.76 *µ*mol/mg for SiGdNP @ D-1 and SiGdNP @ N-1. The longitudinal relaxivity (*r*_1_) increased from 14.3 mM^−1^·s^−1^ for SiGdNP to 18.0 mM^−1^·s^−1^ for SiGdNP @ D-1 or 18.2 mM^−1^·s^−1^ for SiGdNP @ N-1 at 37°C at 60 MHz (Table 1). This might be due to an increase in rotational correlation time resulting from the grafting of a ligand and/or simply the removal of less stable particles or small fragments in the starting population of particles. Meanwhile, the ratio *r*_2_/*r*_1_ stayed around 1.4 (1.36 for SiGdNP vs. 1.42 for both SiGdNP @ D-1 and SiGdNP @ N-1) indicating that the efficiency of the NPs as positive contrast agents was maintained. Results from elemental analysis also confirmed the increases of other elements, i.e., Si, N, and C compared to Gd (Table 1).

Two other syntheses (SiGdNP @ D-2, SiGdNP @ N-2) with the same ratio of SiGdNP and silanes (molar ratio Gd : chelator = 10 : 1.5) were conducted at lower initial concentrations of reactants (50 mM in Gd of SiGdNP and 8 mM of chelating silanes) and lower incubating temperature (40°C). They have more or less similar characteristics compared to previous particles except the homogeneities of the particles which were improved as indicated by full width at half maximum (FWHM) of NP peaks on chromatograms. This was especially true for NODAGA functionalized particles where FWHM of SiGdNP @ N-1 and SiGdNP @ N-2 were 3.4 min and 2.5 min, respectively (Table S2, Figure 2(c), Table S3, Figures S2, S7(A), and S7(C)). But these subtle differences are not anticipated to change significantly the properties of the particles. Two other formulas with higher ratio of APTES-DOTAGA (SiGdNP @ D-3 molar ratio Gd : chelator = 10 : 2, and SiGdNP @ D-4 molar ratio Gd : chelator = 10 : 4) were tested. The result of Eu titration showed that the amount of grafted free chelators was increased as more chelating silanes were added (Figure S4). The chromatograms of these samples do not show any changes in the NP peaks indicating the preservation of homogeneous populations of NPs (Figure S5(B)). The relaxivities of these samples were very similar to each other and similar to other samples (*r*_1_ ∼ 16-17 mM^−1^·s^−1^ *r*_2_/*r*_1_ ∼ 1.4 at 37°C and 60 MHz) (Table S3).

### 2.2. Radiochemistry

**Figure 3:**
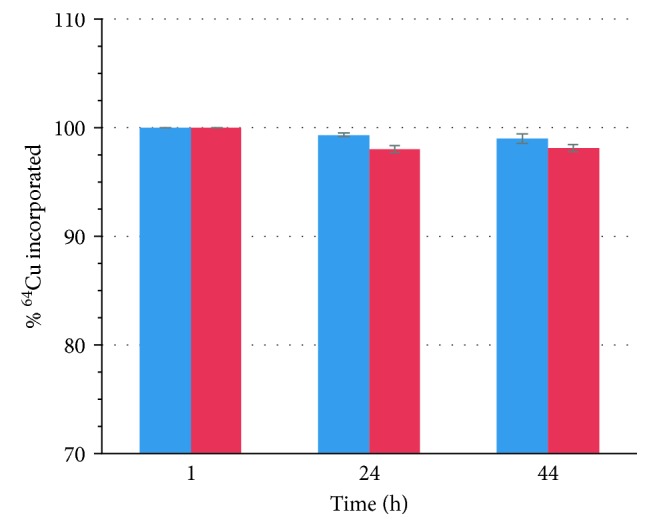
*In vitro* stability of SiGdNP @ N-1(^64^Cu) in plasma (blue) and EDTA solution (red).

SiGdNP @ N-1 was chosen for bimodal MRI/PET imaging experiment due to the presence of gadolinium complexed DOTAGA and free NODAGA on its surface. The latter can complex with ^64^Cu more efficiently. Radiolabeling of the nanoparticles could be easily performed by incubating the nanoparticles with ^64^Cu at 37°C for 45 min. The radiochemical purity was determined to be 100% by ITLC using 0.1 M EDTA as a mobile phase. To further verify the stability of SiGdNP @ N-1(^64^Cu), it was incubated in human plasma and 45 mM EDTA solution over a period of 48 h (Figure 3). The particle retained the radiochemical purity of above 95% in both cases. This experiment confirms the *in vitro* stability of copper chelates on the particle under physiological condition in the presence of competitors that could potentially trigger the decomplexation or trans-chelation. It justifies the use of NODAGA particle for preclinical study.

### 2.3. Simultaneous MRI/PET Imaging Experiments

To evaluate the targeting potential and understand the pharmacokinetics of the nanoparticles, the radiolabeled particles were intravenously injected into mice bearing TS/A tumor. The dose of the nanoparticles was calibrated taking into consideration the amount of the nanoparticles needed for the radiosensitization during the radiotherapy based on previous experience with these nanoparticles [8, 9]. Since each SiGdNP @ N-1(^64^Cu) nanoparticle contains both paramagnetic gadolinium ions and positron emitter ^64^Cu, these NPs appear well suited for simultaneous MRI/PET imaging. Such a combination is very attractive because it allies the high resolution of MRI with the high sensitivity of PET to obtain at the same time accurate anatomical and quantitative information. Although examples of nanoparticles designed for combining MRI and PET were described in the literature, the simultaneous MRI/PET imaging was rarely performed because of the difficulty in developing integrated MRI/PET imaging set-up and the recent arrival on the market of these fused instruments [21]. In many cases, MR images and PET images are collected separately with different animals. These experiments demonstrate that the agent can be followed by MRI or PET but cannot exploit the full potential of a combination between MRI and PET. The main advantage of the simultaneous MR/PET imaging lies in the fact that the biodistribution of SiGdNP @ N-1(^64^Cu) is monitored by both techniques after a single intravenous injection on the same animal. This strategy leads to higher reliability by excluding the problem of different introduced doses, variability in manipulation as well as differences in anatomy, physiology, and position of animals. In this way, this also facilitates the merging of the images since the superimposition of the images does not require complex colocalization method.

**Figure 4:**
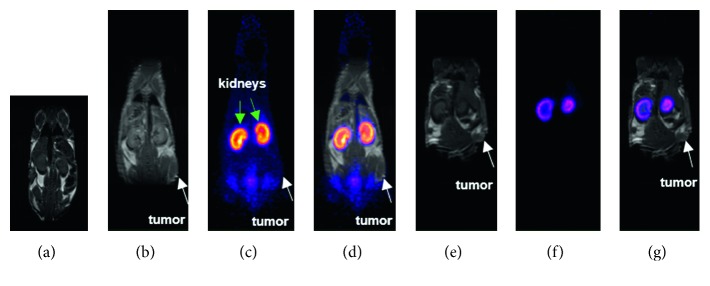
Simultaneous MRI/PET coronal images of mice bearing TS/A tumors (white arrows) after being injected with SiGdNP @ N-1(^64^Cu) nanoparticle. (a) Control *T*_1_-weighted MRI image of a healthy mouse; (b) *T*_1_-MRI; (c) PET, and (d) merged images of tumor bearing mouse 1 h after injection; (e) *T*_1_-MRI; (f) PET and (g) merged images of TS/A of tumor bearing mouse at 24 hours after injection.

**Figure 5:**
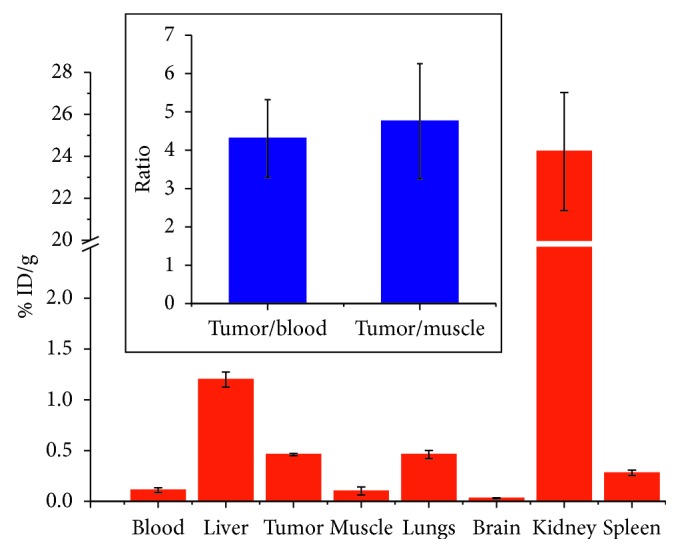
Biodistribution of SiGdNP-NODAGA(^64^Cu) at 24 h after injection in the mice bearing TS/A tumor. Insert: the ratio of signal from tumor tissue over the one from blood or muscle.

As can be seen from Figure 4 (as well as Figures S6 and S7), SiGdNP @ N-1(^64^Cu) provided enhanced contrast in both MRI and PET images. The PET images recorded 1 h and 24 h after intravenous injection clearly indicate that the SiGdNP @ N-1(^64^Cu) freely circulate since the undesirable accumulation in healthy tissues (except in the kidneys) is relatively low 1 h after the injection and almost imperceptible 24 h post injection (pi). It must be pointed out that the presence of nanoparticles in kidneys can be assigned to the renal clearance mechanism since the simultaneous decrease of the positive contrast enhancement and radioactivity in the kidneys is observed between 1 and 24 h pi (Figure S8) [5]. Despite the postfunctionalization which is accompanied by a slight increase of hydrodynamic diameter, the safe biological behavior of SiGdNP is preserved [5]. This highlights the fact that this NP can be cleared via renal excretion and do not suffer from the problem of biopersistence in major organs as is observed with many other inorganic nanoparticles. From these images, it can be deduced that SiGdNP @ N-1(^64^Cu) is well suited for simultaneous MR/PET imaging. This was further illustrated by the biodistribution data at 24 h pi wherein the nanoparticles were observed to be majorly distributed in kidneys (Figure 5). The signal from the kidneys (24.2 ± 2.8% ID/g) was much higher than those in other tissues. However, radioactivity at tumor after 24 h was only 0.46 ± 0.01% ID/g. But this is commonly seen for such ultrasmall nanoparticle which is designed for being rapidly cleared from the body [10, 22, 23]. The modest imaging signal at tumor site 1 h after injection was of more concern (Figures 4(c) and 4(d)). This is contrary to the result we have obtained previously with AGuIX functionalized with Cyanine 5.5 fluorophore in similar TS/A tumor mice model [24]. Probably, the tumors in this study have not grown to sufficient size to manifest disorganization of blood vessels and eventually considerable EPR effect [25].

## 3. Conclusions

We have described a straightforward and simple approach to functionalize ultrasmall silica gadolinium NP along with the physicochemical characterization of the products. Macrocyclic chelating silane precursors have been synthesized by peptide coupling. They can be simply redispersed and grafted on the particle through siloxane bond formation. The amount of free chelators can be tailored according to the starting ratio of precursors. The influence of reaction conditions, i.e., temperature and initial silane concentration, on the homogeneity of the particle was studied. NODAGA functionalized particle was chosen to be labeled with ^64^Cu before being intravenously injected to the mice bearing TS/A tumors. The radiolabeling process was conducted without difficulties. High complexation stability of labeled particle in harsh *in vitro* conditions was verified. MRI/PET images and biodistribution results demonstrate the renal clearability of the particle. However, the accumulation of the particle in the tumor was not significant. This calls for further investigations probably using more mature tumor models in the future.

## 4. Experimental Section

### 4.1. Materials (See S1 for More Details)

The SiGdNP nanoparticles were synthesized by a top-down process described by Mignot et al. [26] and provided by NH TherAguix company (France).

### 4.2. Synthesis of Chelating Silanes

#### 4.2.1. Synthesis of APTES-DOTAGA

DOTAGA(tBu)_4_ (0.9 g, 1.284 mmol) was weighted in 100 mL round bottom flask and was dissolved in 20 mL of DCM with stirring. DIPEA (1.14 mL, 6.55 mmol) was added into the above solution followed by coupling agents viz. HBTU (0.52 g, 1.37 mmol) and HOBt (0.18 g, 1.37 mmol) and the solution was left for stirring at RT for 15 min. APTES (0.3 g, 1.37 mmol) was added to the above solution directly using the 1 mL syringe, and the solution was stirred further at RT for 60 min after which the product formation was confirmed through MS. The above solution was mixed with 50 mL of citric acid solution (pH 2.5–3) in a separating funnel, and the organic layer was recovered. The organic layer was further mixed with 50 mL of 5% NaHCO_3_ in a separating funnel, and the organic layer was recovered. The DCM solution was stirred with 5 g of MgSO_4_ for 10 min and filtered using sintered funnel to receive the dry and clear DCM solution. Organic phase was evaporated under vacuum at 30°C to get a viscous brownish residue, as an intermediate product (1.05 g, 78%). The intermediate formation was verified using HRMS (High resolution Mass Spectrometry), ^1^H, ^13^C NMR, and elemental analysis.

DOTAGA(tBu)_4_-APTES (1 g) was weighed into a 100 mL round bottom flask and was mixed with 5 mL of concentrated HCl (∼12 M) and stirred for 10 min. Later, the acid was evaporated under vacuum at 35°C in 5–15 min to get a dried residue. The dried residue was dissolved in 10 mL of water and lyophilized to get a light brown colored powder (850 mg, 81%). The product was verified using HRMS, ^1^H NMR and, elemental analysis.

#### 4.2.2. Synthesis of APTES-NODAGA

NODAGA(tBu)_3_ (1.0 g, 1.84 mmol) was weighted in 100 mL round bottom flask and was dissolved in 20 mL of DCM under the hood with stirring. DIPEA (1.3 mL, 7.5 mmol) was added into the above solution followed by coupling agents viz. HBTU (1.0 g, 1.95 mmol) and HOBt (1.0 g, 1.95 mmol), and the solution was left for stirring at RT for 15 min. APTES (0.43 g, 1.95 mmol) was added to the above solution directly using the 1 mL syringe, and the solution was stirred further at RT for 60 min after which the product formation was confirmed through MS. The solution was mixed with 50 mL of citric acid solution (pH 2.5–3) in a separating funnel, and the organic layer was recovered. The organic layer was further mixed with 50 mL of 5% NaHCO_3_ in a separating funnel, and the organic layer was recovered. The DCM solution was stirred with 5 g of MgSO_4_ for 10 min and filtered using sintered funnel to receive the dry and clear DCM solution. Organic phase was evaporated under vacuum at 30°C to get a viscous brownish residue, as an intermediate product (1.5 g, 99%). The intermediate formation was verified using HRMS, ^1^H, ^13^C NMR, and elemental analysis.

NODAGA(tBu)_3_-APTES was weighted into a 100 mL round bottom flask and was mixed with 5 mL of concentrated HCl (∼12 M) and stirred for 10 min. Later, the acid was evaporated under vacuum at 35°C in 5–15 min to get a dried residue. The dried residue was dissolved in 10 mL of water and lyophilized to get a light brown-colored powder (1.2 g, 96%). The product was analyzed using HRMS, ^1^H NMR, and elemental analysis.

### 4.3. Characterization of Chelating Silanes (See S2 for More Details)

#### 4.3.1. Intermediate Product (DOTAGA(*t*-Bu)_4_-APTES)

HRMS: calculated for C_44_H_85_N_5_O_12_Si: 926.585 [M+Na]^+^; obtained: m/z = 926.584 [M+Na]^+^ (Figure S9)

^1^H NMR (500 MHz, CDCl_3_): *δ* 0.4–0.7 (m, 2H), 0.7–0.8 (m, 1H), 1.0 (dd, *J* = 9.0, 6.7 Hz, 1H), 1.1–1.2 (m, 9H), 1.3–1.5 (m, 32H), 1.5 (p, *J* = 7.8 Hz, 2H), 1.7 (d, 1H), 1.9–2.1 (m, 1H), 2.1–2.3 (m, 1H), 2.4–3.4 (m, 29H), 3.5–3.7 (m, 1H), 3.7–3.8 (m, 4H).

^13^C NMR (126 MHz, CDCl_3_): *δ* 7.5, 7.8, 18.3, 20.4, 23.5, 25.9, 26.8, 27.8, 27.8, 27.9, 27.9, 27.9, 28.2, 28.3, 29.7, 33.0, 38.6, 42.1, 47.6, 49.8, 58.4, 63.6, 80.8, 82.3, 171.1, 173.2.

Elemental analysis:

Calculated for C_44_H_85_N_5_O_12_Si·0.9HPF_6_ (%): C: 51.03, H: 8.36, N: 6.76.

Observed (%): C: 51.86, H: 8.91, N: 8.38.

#### 4.3.2. APTES-DOTAGA

HRMS: calculated for C_22_H_41_N_5_O_12_Si: 596.259 [M+H]^+^; obtained: m/z = 596.261 [M+H]^+^; 618.242 [M+Na]^+^ (Figure S10).

^1^H NMR (500 MHz, D_2_O): *δ* 0.5–0.8 (m, 2H), 1.2–1.3 (m, 1H), 1.4–1.6 (m, 1H), 1.6–1.8 (m, 1H), 1.8–2.2 (m, 1H), 2.3–4.5 (m, 26H).

Elemental analysis:

Calculated for C_22_H_41_N_5_O_12_Si·HPF_6_·2HCl (%): C: 32.44, H: 5.44, N: 8.60.

Observed (%): C: 32.17, H: 6.54, N: 9.39.

#### 4.3.3. Intermediate Product (NODAGA(*t*-Bu)_3_-APTES)

HRMS: calculated for C_36_H_70_N_4_O_10_Si: 747.493 [M + H]^+^; obtained: m/z = 747.493 [M + Na]^+^ (Figure S11)

^1^H NMR (500 MHz, CDCl_3_): *δ* 0.5–0.6 (m, 2H), 1.0–1.1 (m, 1H), 1.1–1.2 (m, 7H), 1.4 (d, *J* = 7.2 Hz, 25H), 1.5–1.6 (m, 2H), 1.8 (d, *J* = 8.8 Hz, 1H), 1.9–2.1 (m, 1H), 2.2–2.5 (m, 2H), 2.6–3.3 (m, 22H), 3.7–3.8 (m, 4H), 6.2 (d, 1H).

^13^C NMR (126 MHz, CDCl_3_): *δ* 7.8, 18.3, 18.4, 23.1, 26.2, 28.1, 28.2, 28.3, 29.7, 33.4, 38.6, 42.0, 53.7, 55.9, 58.4, 80.8, 165.7, 171.7, 172.8.

Elemental analysis:

Calculated for C_36_H_70_N_4_O_10_Si·0.6HPF_6_ (%): C: 51.81, H: 8.53, N: 6.71.

Observed (%): C: 52.58, H: 9.13, N: 8.85.

#### 4.3.4. APTES-NODAGA

HRMS: calculated for C_18_H_34_N_4_O_10_Si: 495.211 [M + H]^+^; obtained: m/z = 495.212 [M + H]^+^; 517.191 [M + Na]^+^ (Figure S12).

^1^H NMR (500 MHz, D_2_O): *δ* 0.41–0.80 (m, 2H), 0.93–1.28 (m, 2H), 1.39–1.59 (m, 2H), 1.84–2.18 (m, 2H), 2.27–2.46 (m, 2H), 2.73 (s, 5H), 2.97–3.18 (m, 5H), 3.19–3.45 (m, 7H), 3.48–3.75 (m, 1H), 3.79–4.10 (m, 4H).

Elemental analysis:

Calculated for C_18_H_34_N_4_O_10_Si·HPF_6_·HCl (%): C: 31.93, H: 5.36, N: 8.28.

Observed (%): C: 31.74, H: 6.64, N: 8.81.

### 4.4. Grafting of APTES-DOTAGA or APTES-NODAGA on SiGdNP

For SiGdNP @ D-1 and SiGdNP @ N-1, 250 *µ*mol in Gd of SiGdNP was dispersed in water to obtain [Gd^3+^] = 200 mM for 1 h leading to a pH of the solution of 7.4. 29.8 mg (40 *µ*mol) of APTES-DOTAGA or 24.7 mg (40 *µ*mol) of APTES-NODAGA was dissolved in water. pH of the solution was adjusted to 9 by adding NaOH. This solution was left under stirring for 1 h before water was filled to obtain [chelating silane] = 100 mM. It was then gradually added to the SiGdNP solution under stirring at room temperature before water was added to obtain [Gd^3+^] = 100 mM and [chelating silane] = 15 mM. The mixture was left stirring for 1 h before adjusting pH to 4.5. It was heated at 80°C and left under stirring overnight.

For SiGdNP @ D-2, SiGdNP @ D-3, SiGdNP @ D-4, and SiGdNP @ N-2, similar protocol was applied with some modifications: [Gd^3+^] was decreased to 100 mM. 29.8 mg (40 *µ*mol) or 37.2 mg (50 *µ*mol) or 74.5 mg (100 *µ*mol) of APTES-DOTAGA or 24.7 mg (40 *µ*mol) of APTES-NODAGA were used, respectively. The concentration of chelating silane solutions before being added in NP solutions was decreased to 50 mM. At the end, they were heated at 40°C overnight. Detailed parameters can be found in Table S1.

Then each solution was purified with tangential filtration through Vivaspin membranes (MWCO = 3 kDa). The pH of the solution should be adjusted to 2 by adding HCl solutions before the purification. The solution was centrifuged until half of the volume remains. This step was repeated by filling the tubes with hydrochloric acid (HCl) solution 10^−2^ M and centrifuging again for at least 50 purification factor (purification factor = starting volume/end volume). Then, the solution was filtered through 0.2 *µ*m membrane to remove dust and large impurities. Finally, the solution was freeze-dried for long-term storage using a Christ Alpha 1-2 lyophilisator.

### 4.5. Characterization of Functionalized Nanoparticles (See S3 for More Details)

#### 4.5.1. Dynamic Light Scattering (DLS) and Zeta Potential

Hydrodynamic diameter distribution of the nanoparticle was measured by DLS with a Zetasizer Nano-S (633 nm He-Ne laser) from Malvern Instruments (USA).

#### 4.5.2. Infrared Spectroscopy

The IR spectra of dry samples were acquired on an IRAffinity-1, Shimadzu with an ATR platform by applying the attenuated total reflection Fourier transform infrared (ATR-FTIR) spectroscopy from 400 to 4000 cm^−1^.

#### 4.5.3. High-Performance Liquid Chromatography (HPLC)

Gradient HPLC analysis was done by using the Shimadzu Prominence series UFLC system with a CBM-20A controller bus module, a LC-20AD pump, a CTO-20A column oven, and a SPD-20A UV-vis detector.

#### 4.5.4. Phosphorescence Spectroscopy

Phosphorescence measurements were carried out using a Varian Cary Eclipse fluorescence spectrophotometer, in the resolved time mode.

### 4.6. Relaxivity Measurement

Relaxivity measurements were performed on a Bruker® minispec mq60NMR analyzer (Bruker, USA) at 37°C at 1.4 T (60 MHz).

#### 4.7. Elemental Analysis

The contents of elements in SiGdNP @ D-1 and SiGdNP @ N-1 were analyzed by FILAB SAS., Dijon, France. Elemental analyses enabled the determination of the contents of C and N, while inductively coupled plasma-optical emission spectrometry (ICP-OES) was used to determine the contents of Gd and Si.

For other samples, the determination of the content of Gd was performed in the Institut Lumière Matière, Lyon by ICP-OES (with a Varian 710-ES spectrometer).

### 4.8. Radiochemistry and the Stability of Radiolabeled NPs

Radiolabeling: SiGdNP @ N-1 (3.5 mg) was dispersed into 12.9 *µ*L of 1 M, AcONH_4_ buffer pH 5.8, and 12.9 *µ*L of ^64^Cu (7 MBq) in a minitube. The nanoparticle dispersion was incubated at 37°C for 30 min. The radiochemical purity was established by ITLC using 0.1 M EDTA as a mobile phase.

Plasma Stability: 25 *µ*L of the radiolabeled dispersion of nanoparticles was diluted with 45 *µ*L of PBS. 10 *µ*L of the resulting solution was mixed with 100 *µ*L of human plasma in a Eppendorf tube. This tube was incubated at 37°C for 48 h, and aliquots were withdrawn at 24 h and 48 h to test the stability of the radiolabeled nanoparticles using ITLC.

EDTA challenge stability: 25 *µ*L of the radiolabeled dispersion of nanoparticles was diluted with 45 *µ*L of PBS. 10 *µ*L of the resulting solution was mixed with 100 *µ*L of a buffer containing EDTA (50 mM) and HEPES (0.5 M) pH 7 in a minitube. This tube was incubated at 37°C for 48 h, and aliquots were withdrawn at 24 h and 48 h to test the stability of the radiolabeled nanoparticles using ITLC.

The analyses were performed in triplicate wherein the radiolabeled NPs were incubated in plasma (*n*=3) and EDTA challenge solution (*n*=3) separately in Eppendorf tubes. The samples were aliquoted from the same tubes at 24 h and 48 h and analyzed by ITLC. The values have been reported as average with standard deviation (*n*=3).

Radiochromatograms were carried out with a Raytest miniGITA-Star *γ* Radiochromatography (Raytest, Straubenhardt, Germany) or with a Bioscan AR-2000 radio-TLC Imaging Scanner (Bioscan Inc., Washington, DC).

### 4.9. Animal Studies

All animal studies were conducted in accordance with the legislation on the use of laboratory animals (directive 2010/63/EU) and were approved by accredited Ethical committee (C2ea Grand Campus no. 105).

Female NMRI nude mice (6–8 weeks, Janvier Labs, France) were subcutaneously injected with 1.10^7^ TS/A cells (murine mammary adenocarcinoma).

Mice were maintained in ventilated housing units under controlled conditions of temperature (22 ± 2°C) and photoperiod (12 h light/12 h dark) with free access to food and drink.

Three to five weeks after tumor cells injection, TS/A tumor bearing-mice (*n*=3) were given 10 mg of SiGdNP @ N-1(^64^Cu) in PBS corresponding to radioactivity of 10.2 ± 0.3 MBq by intravenous injection. Size of the tumors varies from 41 to 157 mm^3^ with a mean value of 105 mm.

For biodistribution, at the end of 24 h, animals were euthanized by isoflurane anesthesia followed by pentobarbital overdose. Blood, tumor, and organs were collected, weighed, and radioactivity in these samples was measured with a scintillation gamma-counter (Cobra 4180, Perkin Elmer, Waltham, MA, USA).

### 4.10. MRI-PET Imaging and Biodistribution Measurement

Simultaneous MRI-PET imaging was performed at 1 and 24 hours after the injection of the radiolabeled nanoparticles on a fully integrated system (MR Solutions, Guilford, UK) consisting of a 7T dry magnet (Powerscan MRS-7024-PW) coupled with a SiPM dual-ring PET-I-802 insert.

Mice were anaesthetized with isoflurane (2% in oxygen) and positioned in a dedicated heating cradle. MRI and PET acquisitions were performed simultaneously. Animal respiration was monitored with abdominal pressure sensor and dedicated software (PC Sam, SAII, Stony Brook, US).

List-mode data were collected in the PET system during 30–60 minutes. Images were reconstructed with the 3D ordered subset expectation maximization (OSEM) algorithm implemented in the system using 2 iterations, 32 subsets, and an isotropic voxel size of 0.28 mm. An energy window of 250–750 keV and a coincidence time window of 8 ns were applied to the list-mode data. The algorithm takes into account normalization and random and decay corrections. No attenuation correction was applied.

*T*_1_- and *T*_2_-weighted fast spin echo MR images were acquired with respiratory gating and sequences in axial and coronal plans. *T*_1_ sequences were acquired with time of repetition (TR) of 1000 ms, time of echo (TE) of 11 ms. *T*_2_ sequences were acquired with TR of 4000 ms, TE of 45 ms. Following parameters were used for both *T*_1_ and *T*_2_: flip angle of 90°, 4 signal averages, 1 mm slice thickness, 0.15 × 0.15 mm^2^ transaxial pixel size, and 256 × 256 pixels matrix.

Finally, the PET-MR fusion image was obtained using VivoQuant (Invicro, Boston, US). Each scan was then visually interpreted.

For the gamma counting of the organs, the counter was cross-calibrated to the dose calibrator used to measure the injected dose, and the linearity range was determined for all the geometries used in *ex vivo* counting. Data were then converted to percentage of injected dose and to percentage of injected dose per gram of tissue.

## Figures and Tables

**Table 1:** Summary of characterizations of NPs before and after functionalization with chelating silanes.

Properties	Method(s)	SiGdNP	SiGdNP @ D-1	SiGdNP @ N-1
*D*_H_ (nm)	DLS	4.2 ± 0.8	4.5 ± 0.9	4.5 ± 0.9
Zeta potential (mV)	Zetametry	+8.2 (pH 7.0)	−8.3 (pH 7.0)	−13.7 (pH 7.0)
Free COOH band	IR	No	Yes	Yes
Retention time (min)	HPLC (295 nm)	13.6	15.3	14.7
Free chelator content (*µ*mol/mg)	Eu^3+^ titration	0.025	0.10	0.10
Gd content (*µ*mol/mg)	ICP-OES	0.890	0.763	0.763
*r*_1_ (mM^−1^·s^−1^) (37°C, 60 MHz)	Relaxometry	14.33	17.97	18.17
*r*_2_/*r*_1_	Relaxometry	1.36	1.42	1.42
Gd : Si : N : C	Elemental analysis	1.0 : 4.8 : 6.0 : 25.3	1.0 : 6.5 : 7.3 : 30.7	1.0 : 6.1 : 7.2 : 30.8

## Data Availability

The data used to support the findings of this study are included within the article and the supplementary information file. Any other data are available from the corresponding author upon request.

## Supplementary Materials

Supplementary Materials Supplementary materials contains an experimental section (S1) describing the different chemicals products used for the synthesis and a description of the cheracterization methods of the silanes (S2) and of the functionalized nanoparticles (S3). Two figures are present (S9 and S10) to describe the MRI and the PET/MRI images after intravenous administration of the radiolabeled NPs.
