# Supplementary material for: Functionalization of Gadolinium Chelates Silica Nanoparticle through Silane Chemistry for Simultaneous MRI/64Cu PET Imaging
Source: Contrast Media Mol Imaging. 2018 Nov 1;2018:7938267. doi: 10.1155/2018/7938267 (PMC6236700; doi:10.1155/2018/7938267)
Supplement: Supplementary Materials — contains an experimental section (S1) describing the different chemicals products used for the synthesis and a description of the cheracterization methods of the silanes (S2) and of the functionalized nanoparticles (S3). Two figures are present (S9 and S10) to describe the MRI and the PET/MRI images after intravenous administration of the radiolabeled NPs. [file 7938267.f1.docx]

Supporting Information for

Functionalization of Gadolinium Chelates Silica Nanoparticle

through Silane Chemistry for Simultaneous MRI/PET Imaging

###### Table of contents

[Experimental Section 1](#_Toc507610773)

[S1. Materials 1](#_Toc507610774)

[S2. Characterization of chelating silanes 2](#_Toc507610775)

[S3. Characterization of functionalized nanoparticles 2](#_Toc507610776)

[S3.1. Dynamic light scattering (DLS) and zeta potential 2](#_Toc507610777)

[S3.2. Infrared spectroscopy 2](#_Toc507610778)

[S3.3. High Performance Liquid Chromatography (HPLC) 2](#_Toc507610779)

[S3.4. Titration by Eu phosphorescence 2](#_Toc507610780)

[S3.5. Relaxivity measurement. 2](#_Toc507610781)

[S3.6. Elemental analysis. 3](#_Toc507610782)

###### Experimental Section

###### Materials

For the synthesis of chelating silanes, all the chemicals used to synthesize the compounds were purchased from Acros, unless otherwise stated. Following is the list of reactants and their respective vendors: HOBt: Fluorochem; HBTU: Iris BiotechTris; DIPEA, NAHCO_3_: Alfa Aesar; DCM (Dichloromethane): VWR. 5-(tert-butoxy)-5-oxo-4-(4,7,10-tris(2-(tert-butoxy)-2-oxoethyl)-1,4,7,10 tetraazacyclododeca- n-1-yl) pentanoic acid ((*t-*Bu)_4_DOTAGA) and 4-(4,7-bis(2-(tert-butoxy)-2-oxoethyl)-1,4,7-triazacyclononan-1-yl)-5-(tert-butoxy)-5-oxopentanoicacid ((*t-*Bu)_3_NODAGA) were obtained from CheMatech® and used without further purification. ^64^Cu was obtained as Copper (^64^Cu) chloride in 0.1 N Hydrochloric acid (Cuprymina 925 MBq/mL) from SPARKLE S.r.l, Italy.

Copper sulfate pentahydrate (CuSO_4_.5H_2_O, 98%) was purchased from Merck (France). Hydrochloric acid (HCl, 37%) was purchased from VWR Chemicals BDH Prolabo (France). Sodium hydroxide pellets (NaOH, ≥ 98%) were purchased from Sigma-Aldrich Chemicals (France). Solutions of hydrochloric acid and sodium hydroxide in water at different concentrations from 2 M to 10^-4^ M were prepared to adjust pH of solutions. Aminopropyltriethoxysilane (H_2_N(CH_2_)_3_-Si(OC_2_H_5_)_3_, APTES, 99%), Trifluoroacetic acid (TFA) analytical standard, glacial acetic acid for preparing buffer at pH 5 were purchased from Sigma-Aldrich Chemicals (France). Gadolinium chloride hexahydrate (GdCl_3_.6H_2_O, 99.999%) and europium chloride hexahydrate (EuCl_3_.6H_2_O, 99.999%) were purchased from Metal Rare Earth Limited (China). Acetonitrile for HPLC Plus Grade was purchased from Carlo Erba Reagents (France). Milli-Q water (ρ > 18 MΩ) was used as water source. Vivaspin™ concentrators (MWCO = 3 kDa or 5 kDa) were purchased from Sartorius Stedim Biotech (France). Gd (1000 mg/mL ± 0.2%) ICP single element standard solutions were purchased from Carl Roth (France).

###### Characterization of chelating silanes

**Methods**

The ^1^H and ^13^C NMR spectra were recorded at room temperature or at 330 K. NMR spectra were run on BRUKER Avance 300 and/or 500 spectrometers using per-deuterated solvents as internal standard. Elemental analyses were obtained on EA 1108 CHNS Fisons Instrument. ESI (Electro Spray Ionisation). High resolution and accurate mass measurements (HRMS) were carried out using a Bruker microTOF-Q™ ESI-TOF mass spectrometer.

###### Characterization of functionalized nanoparticles

Dynamic light scattering (DLS) and zeta potential

Measurement was taken on 1 ml of the solution at around 5 – 10 g/l with a single use PMMA cuvette (Carl Roth GmbH, Germany) at 20 °C. Attenuator and position were optimized by the device. Fast mode was enabled to enhance the precision for the measurement of particles with hydrodynamic diameter less than 10 nm.

To determine zeta potential, lyophilized powder was redispersed in water to achieve 100 mg/ml solution and diluted to 10 mg/ml in an aqueous solution containing 5 mM NaCl and adjusted to the desired pH just before each measurement. Zeta potential measurements were recorded at 20 °C within a DTS 1061 folded capillary cell (Malvern Instruments Ltd, USA). The zeta potential (ζ) was automatically calculated from electrophoretic mobility based on the Smoluchowski equation, ν = (εε_0_ζ /η)ζ, where ν is the measured electrophoretic mobility, η is the viscosity, ε is the dielectric constant of the electrolytic solution, ε_0_ ≈ 8.854 x 10^-12^ C^2^N^-1^m^-2^ is the vacuum permittivity.

Infrared spectroscopy

SiGdNP, SiGdNP@D-1 and SiGdNP@N-1 were dissolved in water and adjusted to pH 2 to protonate carboxyl groups. This makes the peak at 1677 cm^-1^ of C=O amide distinguished from the one at 1713 cm^-1^ of C=O carboxyl. The 3 solutions were freeze-dried. IR spectra were acquired with dry powder. Figure S5 shows the IR spectrum of SiGdNP, SiGdNP@D-1 and SiGdNP@N-1. The appearance of peak at 1730 cm^-1^ is an indication of the presence of free carboxyl.^1^

High Performance Liquid Chromatography (HPLC)

The detecting wavelength was set at 295 nm. The column temperature was maintained at 30 °C. Gradient LC elution was carried out with two mobile phases: (A) Milli-Q water/TFA 99.9:0.1 v/v and (B) acetonitrile (CH_3_CN)/TFA 99.9:0.1 v/v. Each time, an amount of 20 μL of sample was loaded to an injection valve and injected into a Jupiter C4 column (150 mm × 4.60 mm, 5 μm, 300 Å, Phenomenex) at a flow rate of 1 mL/min. Then the elution was programmed as followings: 1% of solvent B in 7 min to elute the reactive and fragments, then a gradient from 1% to 90% in 15 min to elute the nanoparticle. The concentration of B was maintained over 7 min. Then, the concentration of solvent B was decreased to 1% over 1 min and maintained during 8 min to re-equilibrate the system for a new analysis. Before the measurement of each sample, a baseline was obtained under the same conditions by injecting Milli-Q water. The purity is calculated by dividing the area under the peak of the particle to the total area under the peaks of the particle and the reactives.

Titration by Eu^3+^ phosphorescence

A series of samples with a certain amount of chelator and an increasing amount of EuCl_3_ was prepared in acetate buffer pH 5. These series of samples were incubated at 80^o^C for 48 h before the measurement. Scan mode was used with parameters as following: excitation wavelength = 395 nm, excitation slit = 20 nm, emission slit = 10 nm, averaging time 0.1 s, data interval 1 nm, total decay time 3 ms, number of flash 1, delay time 0.1 ms, gate time 2 ms, no excitation filter, emission filter at 430 – 1100 nm, 800 V voltage. The titration curves were drawn from the emission at 594 nm of Eu^3+^ ions. In some measurements, the areas under the peak at 594 nm were used instead of single value to have higher precision. The content (µmol/mg) of free chelators can be calculated from its molar concentration (µmol/L) determined by the assay and the initial mass concentration (mg/L) of the analyzed sample.

Relaxivity measurement.

Samples were measured at a specific Gd^3+^ concentration (mM), measured from ICP-MS. The longitudinal relaxation time T_1_ and the transverse relaxation time T_2_ (s) were measured. Then the relaxivities r_i_ (s^−1^ .mM^−1^) (i = 1, 2) were obtained according to the following formula:

$$\left( \frac{1}{T_{i}} \right)measured= \left( \frac{1}{T_{i}} \right)water+r_{i}\left[ {Gd}^{3+} \right]$$

$$T_{1 water}\approx3500 \left( ms \right); T_{2 water}\approx3390 \left( ms \right)$$

$$i=1 or 2$$

Elemental analysis.

For samples measured in ILM, the solution of particles at an estimated concentration in Gd of 10 ppm was digested for 3h in 4-5 mL of aqua regia (HNO_3_ 67% mixed with HCl 37% (1/2; v/v)) at 80 °C. Subsequently, the mixture was diluted to estimated 100, 200 and 400 ppb at precisely 50 mL with HNO_3_ 5% (v/v). These solutions were filtered through 0.2 µm membrane before being analyzed. Calibrated samples were prepared from 1000 ppm Gd standard solution by successive dilutions with HNO_3_ 5% (w/w). The result was the average of the three samples at presumably 100, 200 and 400 ppb.

Table S1. Summary of type of silane, starting concentrations of components and temperature used in different formulas

| Formula code | Type of silane | [SiGdNP] (mM in Gd) | [Silane] (mM) | Temperature (^o^C) |
| --- | --- | --- | --- | --- |
| SiGdNP@D-1 | APTES-DOTAGA | 100 | 15 | 80 |
| SiGdNP@D-2 | APTES-DOTAGA | 50 | 8 | 40 |
| SiGdNP@D-3 | APTES-DOTAGA | 50 | 10 | 40 |
| SiGdNP@D-4 | APTES-DOTAGA | 50 | 20 | 40 |
| SiGdNP@N-1 | APTES-NODAGA | 100 | 15 | 80 |
| SiGdNP@N-2 | APTES-NODAGA | 50 | 8 | 40 |

A)


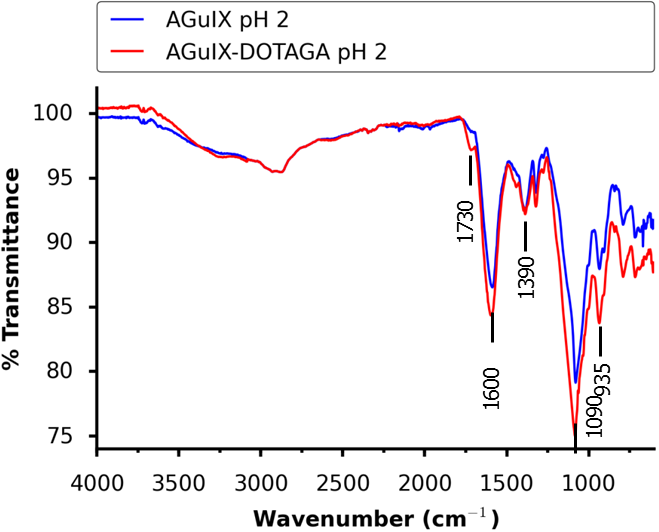


SiGdNP

SiGdNP@D-1


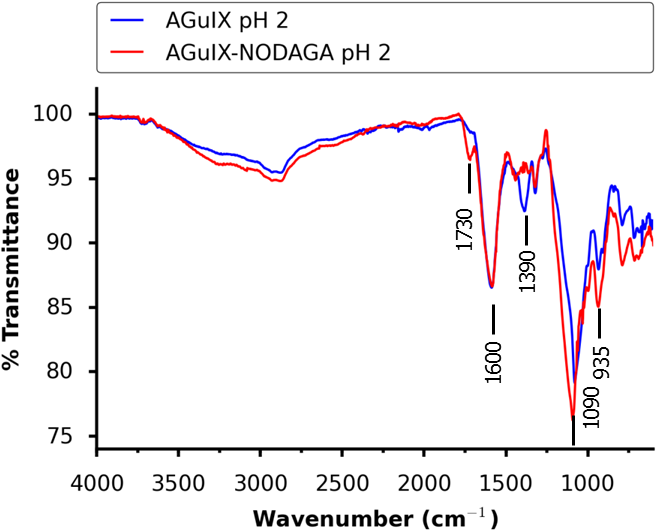


SiGdNP

SiGdNP@N-1

B)

Figure S1. Infrared spectra of:

A) SiGdNP before functionalized (blue) and SiGdNP@D-1 (after functionalized with DOTAGA) (red);

B) SiGdNP before functionalized (blue) and SiGdNP@N-1 (after functionalized with NODAGA) (red).

Figure S1. Infrared spectra of:

A) SiGdNP before functionalized (blue) and SiGdNP@D-1 (after functionalized with DOTAGA) (red);

B) SiGdNP before functionalized (blue) and SiGdNP@N-1 (after functionalized with NODAGA) (red).

Table S2. Full summary of characterizations of NPs before and after being functionalized with chelating silanes at 80^o^C incubation

| Properties | Method(s) | SiGdNP | SiGdNP@D-1 | SiGdNP@N-1 |
| --- | --- | --- | --- | --- |
| D_H_  (nm) | DLS | 4.2 ± 0.8 | 4.5 ± 0.9 | 4.5 ± 0.9 |
| Zeta potential (mV) | Zetametry | + 8.2  (pH 7.0) | - 8.3  (pH 7.0) | - 13.7  (pH 7.0) |
| Free COOH band | IR | No | Yes | Yes |
| Retention time (min) | HPLC (295 nm) | 13.6 | 15.3 | 14.7 |
| FWHM (min) | HPLC (295 nm) | 2.0333 | 2.9917 | 3.3833 |
| Purity (%) | HPLC (295 nm) | 85.1 | 95.1 | 96.4 |
| Free chelator content (µmol/mg) | Eu^3+^ titration | 0.025 | 0.1 | 0.1 |
| % chelator added |  | - | 15 | 15 |
| % chelator grafted | Eu^3+^ titration | - | 11 | 11 |
| Gd content (µmol/mg) | ICP-OES | 0.890 | 0.763 | 0.763 |
| r_1_ (mM^-1^.s^-1^)  (37^o^C, 60 MHz) | Relaxometry | 14.33 | 17.97 | 18.17 |
| r_2_/r_1_ | Relaxometry | 1.36 | 1.42 | 1.42 |
| Gd : Si : N : C | Elemental analysis | 1.0 : 4.8 : 6.0 : 25.3 | 1.0 : 6.5 : 7.3 : 30.7 | 1.0 : 6.1 : 7.2 : 30.8 |
| Estimated formula* | Calculated | Gd_1.0_A-D_1.0_A_0.8_T_3.0_ | Gd_1.0_A-D_1.3_A_1.0_T_4.2_ | Gd_1.0_A-D_1.0_A-N_0.3_A_0.7_T_4.0_ |
| Yield (%) (in chelator) |  | - | 24.0 | 26.9 |
| Yield (%) (in Gd) |  | - | 28.6 | 32.0 |
| **A-D: APTES-DOTAGA, A-N : APTES-NODAGA, A: APTES, T: TEOS* | | | | |

Table S3. Summary of characterizations of functionalized NPs in different conditions at 40^o^C incubation

| Properties | Method(s) | SiGdNP@D-2 | SiGdNP@D-3 | SiGdNP@D-4 | SiGdNP@N-2 |
| --- | --- | --- | --- | --- | --- |
| D_H_  (nm) | DLS | 4.0 ± 0.9 | 3.8 ± 1.0 | 3.4 ± 1.0 | 4.0 ± 0.9 |
| Zeta potential (mV) | Zetametry | 0.07  (pH 6.8) | - 16.7  (pH 6.9) | - 18.8  (pH 6.9) | - 12.1  (pH 6.8) |
| Free COOH band | IR | - | - | - | - |
| Retention time (min) | HPLC (295 nm) | 14.7 | 14.8 | 14.9 | 14.6 |
| FWHM (min) | HPLC (295 nm) | 2.4917 | 2.4500 | 2.2500 | 2.5250 |
| Purity (%) | HPLC (295 nm) | 91.7 | 91.7 | 92.1 | 91.6 |
| Free chelator content (µmol/mg) | Eu^3+^ titration | 0.09 | 0.16 | 0.20 | 0.12 |
| % chelator added |  | 15 | 20 | 40 | 15 |
| % chelator grafted | Eu^3+^ titration | 12 | 20 | 28 | 17 |
| Gd content (µmol/mg) | ICP-OES | 0.643 | 0.640 | 0.515 | 0.650 |
| r_1_ (mM^-1^.s^-1^)  (37^o^C, 60 MHz) | Relaxometry | 16.11 | 12.13 | 17.16 | 16.99 |
| r_2_/r_1_  (37^o^C, 60 MHz) | Relaxometry | 1.38 | 1.49 | 1.37 | 1.42 |
| Yield (%) (in chelator) |  | 41.6 | 46.3 | 33.8 | 48.6 |
| Yield (%) (in Gd) |  | 47.5 | 37.0 | 34.8 | 38.9 |

A)

Figure S2. DLS diagrams of:A)

Figure S2. DLS diagrams of:

A) SiGdNP@D-1 (DOTAGA, 80^o^C) (dash-dotted line) and SiGdNP@D-2 (DOTAGA, 40^o^C) (solid line);

B) SiGdNP@D-2 (DOTAGA, 40^o^C, DOTAGA:Gd = 15 :100) (black), SiGdNP@D-3 (DOTAGA, 40^o^C, DOTAGA:Gd = 20 :100) (blue), SiGdNP@D-4 (DOTAGA, 40^o^C, DOTAGA:Gd = 40 :100) (red);

C) SiGdNP@N-1 (NODAGA, 80^o^C) (dash-dotted line) and SiGdNP@N-2 (NODAGA, 40^o^C) (solid line);

Figure S2. DLS diagrams of:

A) SiGdNP@D-1 (DOTAGA, 80^o^C) (dash-dotted line) and SiGdNP@D-2 (DOTAGA, 40^o^C) (solid line);

B) SiGdNP@D-2 (DOTAGA, 40^o^C, DOTAGA:Gd = 15 :100) (black), SiGdNP@D-3 (DOTAGA, 40^o^C, DOTAGA:Gd = 20 :100) (blue), SiGdNP@D-4 (DOTAGA, 40^o^C, DOTAGA:Gd = 40 :100) (red);

C) SiGdNP@N-1 (NODAGA, 80^o^C) (dash-dotted line) and SiGdNP@N-2 (NODAGA, 40^o^C) (solid line);

C)

C)

B)

B)

SiGdNP@N-1

SiGdNP@N-2

SiGdNP@N-1

SiGdNP@N-2

SiGdNP@D-1

SiGdNP@D-2

SiGdNP@D-1

SiGdNP@D-2

SiGdNP@D-2

SiGdNP@D-3

SiGdNP@D-4

SiGdNP@D-2

SiGdNP@D-3

SiGdNP@D-4

SiGdNP@D-1

SiGdNP@D-2

Figure S3. HPLC chromatograms of:SiGdNP@D-1

SiGdNP@D-2

Figure S3. HPLC chromatograms of:

A) SiGdNP@D-1 (DOTAGA : Gd = 1.5 : 10, 80^o^C) (black), SiGdNP@D-2 (DOTAGA : Gd = 1.5 : 10, 40^o^C) (red);

B) SiGdNP@N-1 (NODAGA : Gd = 1.5 : 10, 80^o^C) (black), SiGdNP@N-2 (NODAGA : Gd = 1.5 : 10, 40^o^C) (red).

Figure S3. HPLC chromatograms of:

A) SiGdNP@D-1 (DOTAGA : Gd = 1.5 : 10, 80^o^C) (black), SiGdNP@D-2 (DOTAGA : Gd = 1.5 : 10, 40^o^C) (red);

B) SiGdNP@N-1 (NODAGA : Gd = 1.5 : 10, 80^o^C) (black), SiGdNP@N-2 (NODAGA : Gd = 1.5 : 10, 40^o^C) (red).

SiGdNP@N-1

SiGdNP@N-2

SiGdNP@N-1

SiGdNP@N-2

A)

A)

B)

B)


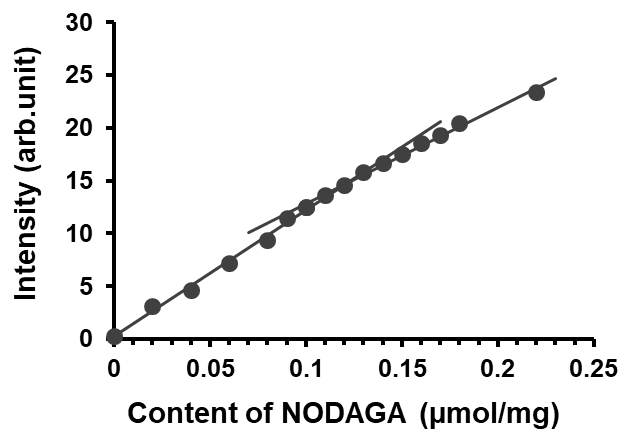


A)

Figure S4. Eu titration curves λ_ex_ = 395 nm, λ_em_ = 594 nm of A) SiGdNP@N-2, B) SiGdNP@D-2, C) SiGdNP@D-3 and D)SiGdNP@D-4A)


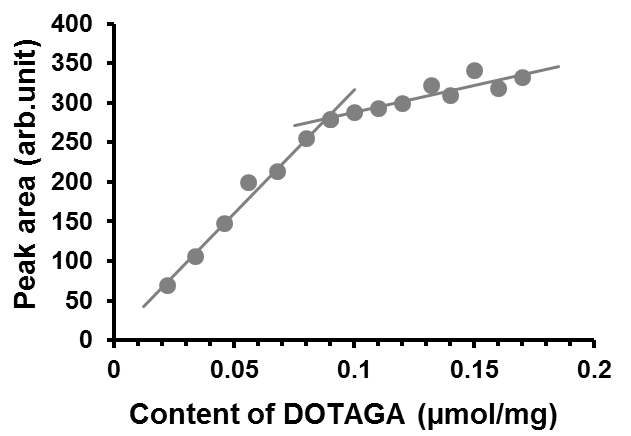

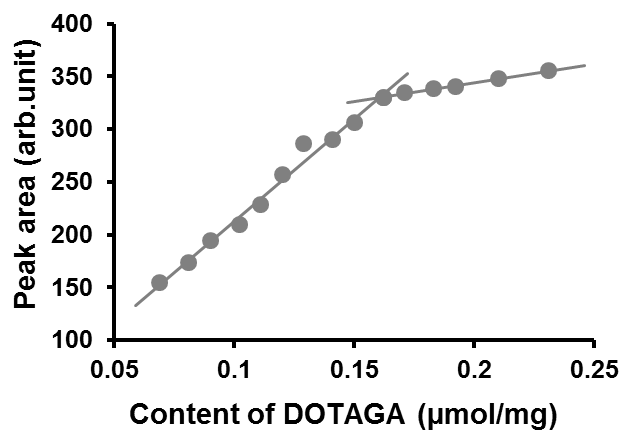

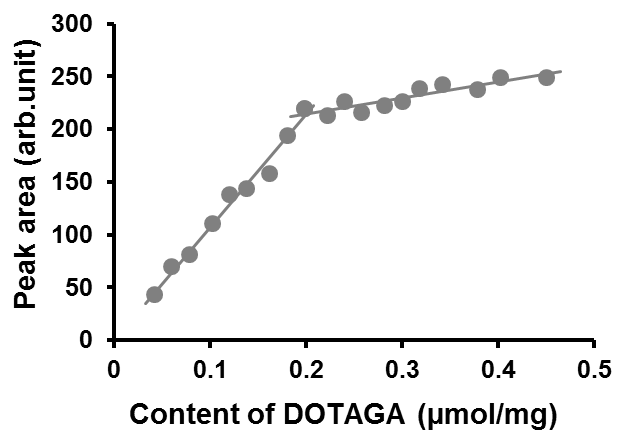


Figure S4. Eu titration curves λ_ex_ = 395 nm, λ_em_ = 594 nm of A) SiGdNP@N-2, B) SiGdNP@D-2, C) SiGdNP@D-3 and D)SiGdNP@D-4

Figure S4. Eu titration curves λ_ex_ = 395 nm, λ_em_ = 594 nm of A) SiGdNP@N-2, B) SiGdNP@D-2, C) SiGdNP@D-3 and D)SiGdNP@D-4

B)

B)

C)

C)

D)

Figure S5. HPLC chromatograms of SiGdNP@D-2 (DOTAGA : Gd = 1.5 : 10) (black), SiGdNP@D-3 (DOTAGA : Gd = 2 : 10) (blue), SiGdNP@D-4 (DOTAGA : Gd = 4 : 10) (red)D)

Figure S5. HPLC chromatograms of SiGdNP@D-2 (DOTAGA : Gd = 1.5 : 10) (black), SiGdNP@D-3 (DOTAGA : Gd = 2 : 10) (blue), SiGdNP@D-4 (DOTAGA : Gd = 4 : 10) (red)

Figure S5. HPLC chromatograms of SiGdNP@D-2 (DOTAGA : Gd = 1.5 : 10) (black), SiGdNP@D-3 (DOTAGA : Gd = 2 : 10) (blue), SiGdNP@D-4 (DOTAGA : Gd = 4 : 10) (red)

SiGdNP@D-2

SiGdNP@D-3

SiGdNP@D-4

SiGdNP@D-2

SiGdNP@D-3

SiGdNP@D-4


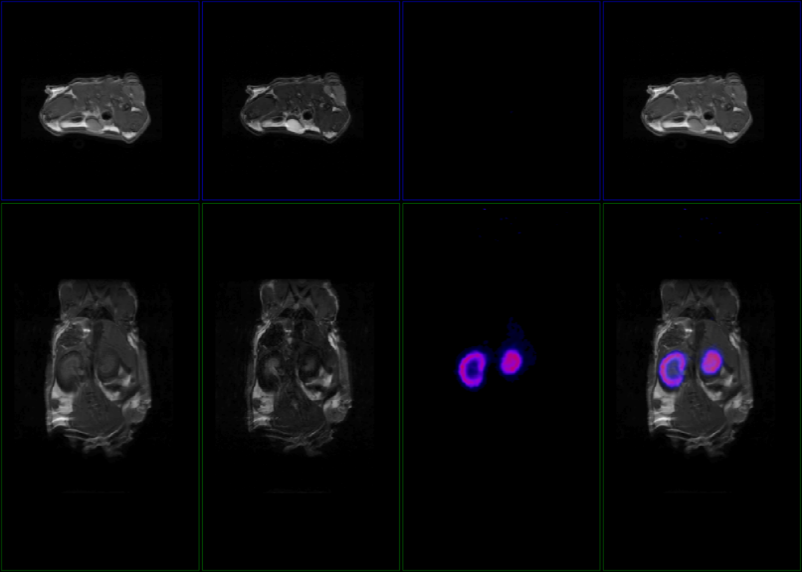

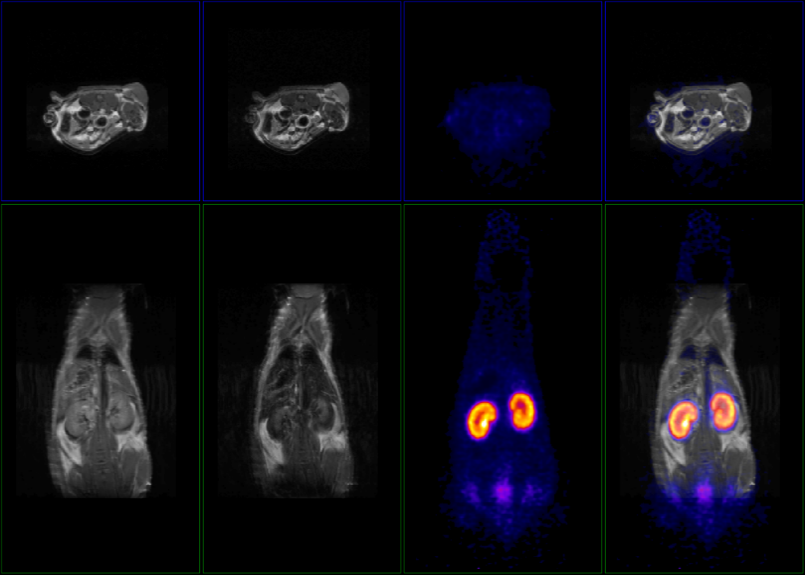


**(A)**

**(A)**

**(B)**

Figure S6. T2 weighted MRI images of tumor bearing mouse (A) at 1 h after injection and (B) at 24 h after injection of SiGdNP@N-1(^64^Cu) nanoparticle.**(B)**

Figure S6. T2 weighted MRI images of tumor bearing mouse (A) at 1 h after injection and (B) at 24 h after injection of SiGdNP@N-1(^64^Cu) nanoparticle.

Figure S6. T2 weighted MRI images of tumor bearing mouse (A) at 1 h after injection and (B) at 24 h after injection of SiGdNP@N-1(^64^Cu) nanoparticle.

**tumor**

**tumor**

**tumor**

**tumor**

**(A)**

**(A)**

**(B)**

**(B)**

**(C)**

**(C)**

**(D)**

**(D)**

**(E)**

**(E)**

**(F)**

**(F)**

**(G)**

**(G)**

**(H)**

Figure S7. Simultaneous MRI/PET axial images of mice bearing TS/A tumors after being injected with SiGdNP@N-1(^64^Cu) nanoparticle: (A) T1 weighted MRI, (B) T2 weighted MRI, (C) PET and (D) merged images (PET/T1-MRI) at 1 h after injection; (E) T1-MRI, (F) T2-MRI, (G) PET and (H) merged images (PET/T1-MRI) at 24 h after injection.**(H)**


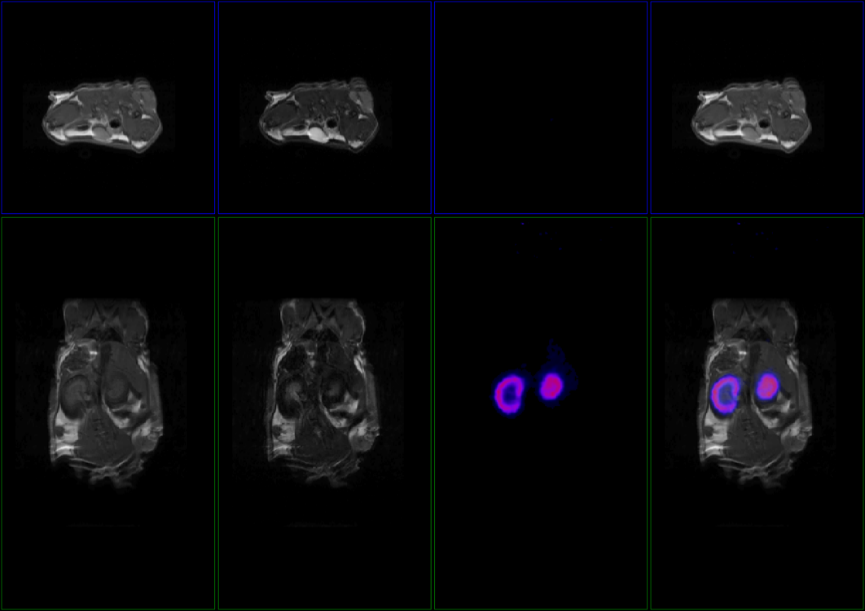

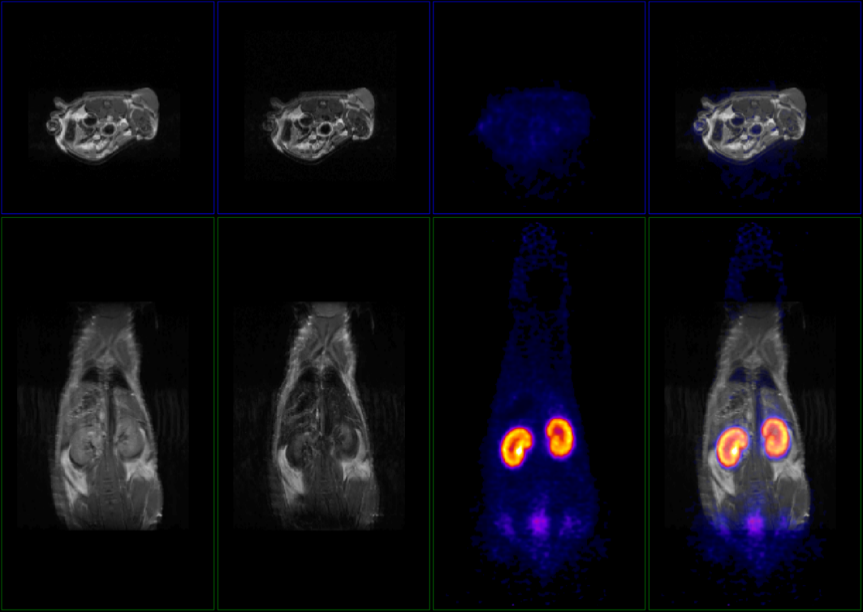


Figure S7. Simultaneous MRI/PET axial images of mice bearing TS/A tumors after being injected with SiGdNP@N-1(^64^Cu) nanoparticle: (A) T1 weighted MRI, (B) T2 weighted MRI, (C) PET and (D) merged images (PET/T1-MRI) at 1 h after injection; (E) T1-MRI, (F) T2-MRI, (G) PET and (H) merged images (PET/T1-MRI) at 24 h after injection.

Figure S8. Kidney to muscle ratio contrast in the kidneys before administration, 1h and 24h after administration


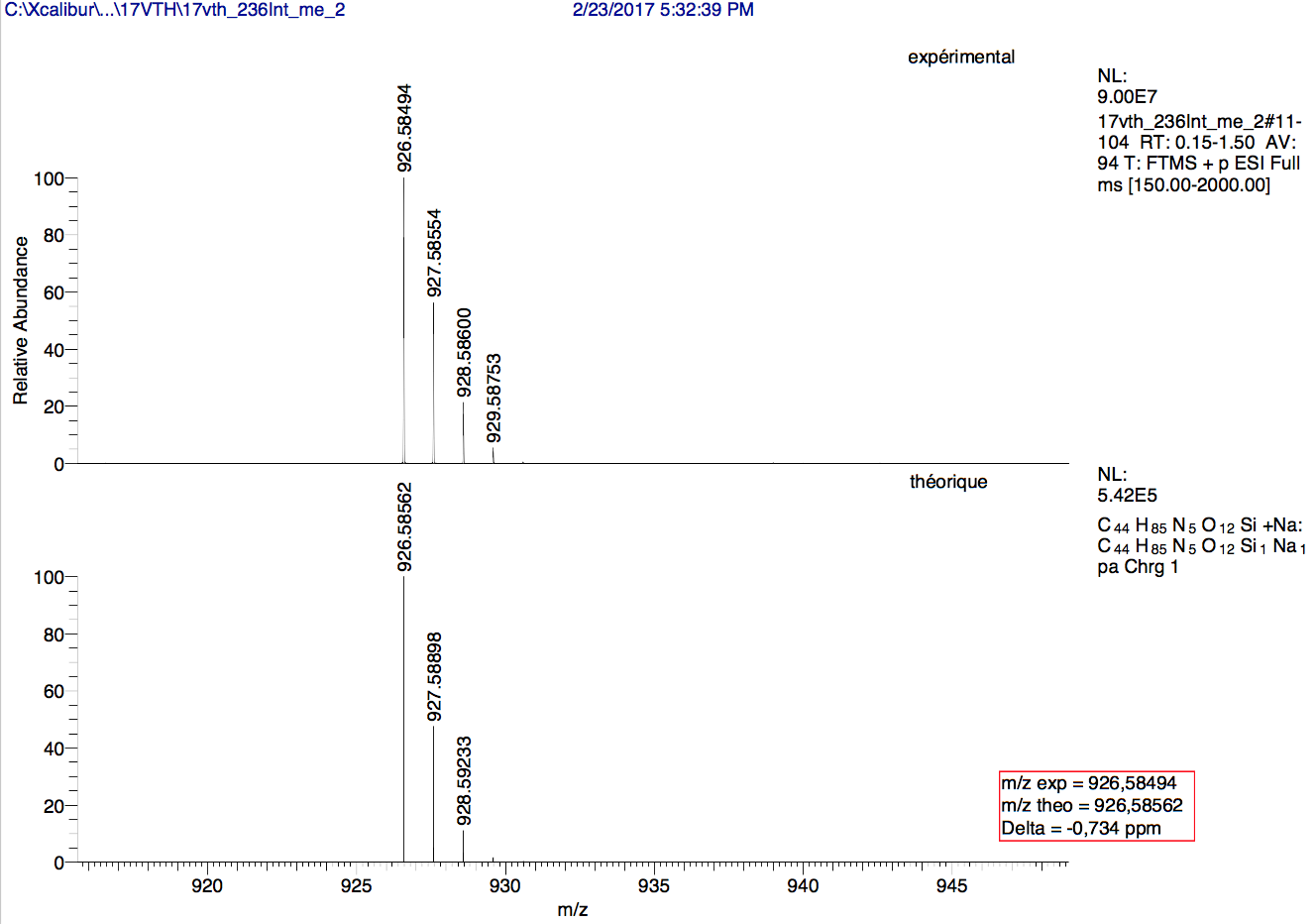


**A)**

**B)**

Figure S9. Mass spectrum of butyl protected APTES-DOTAGA: A) experimental spectrum, B) simulated spectrum


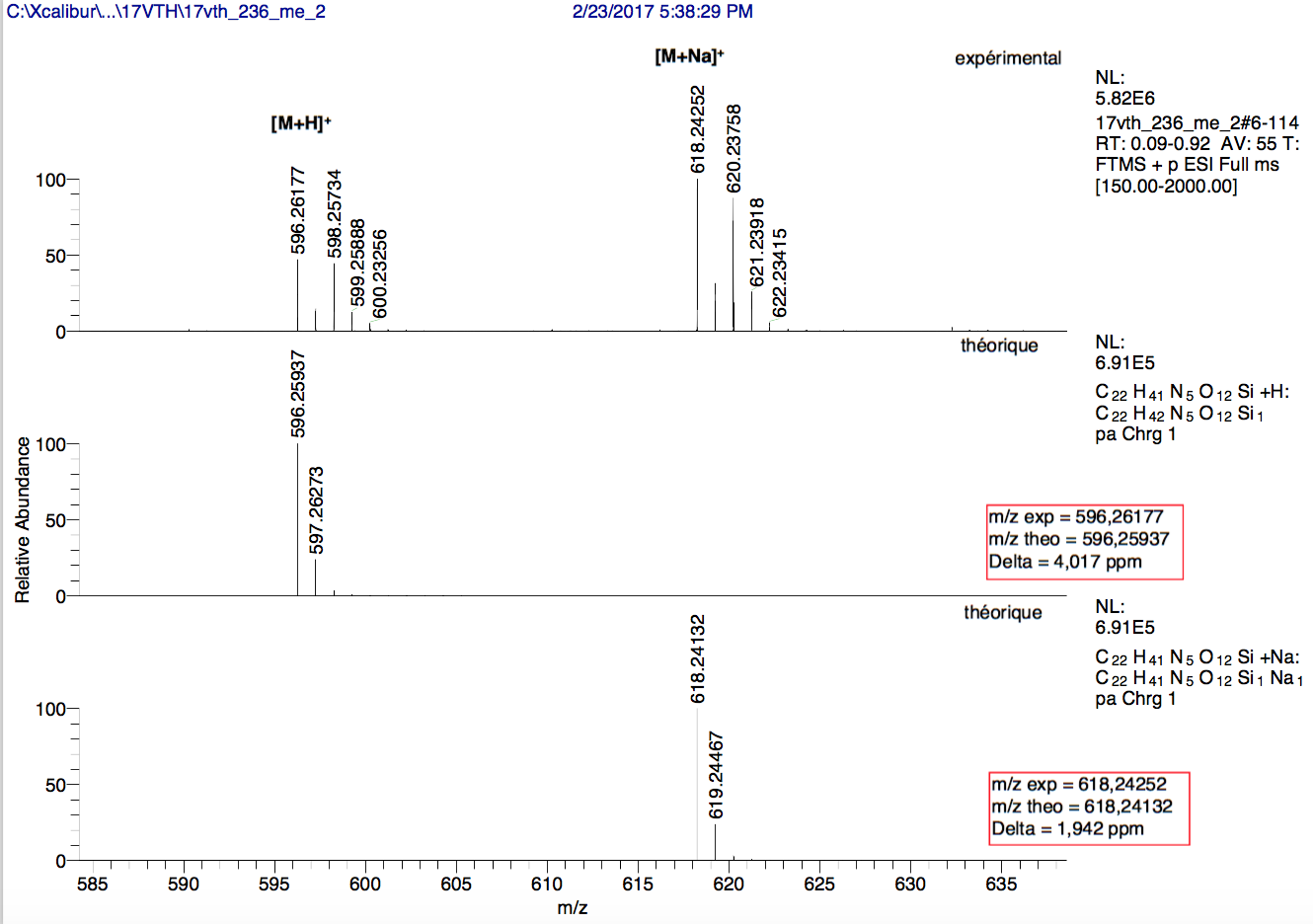


**A)**

**B)**

Figure S10. Mass spectrum of deprotected APTES-DOTAGA: A) experimental spectrum, B) simulated spectrum


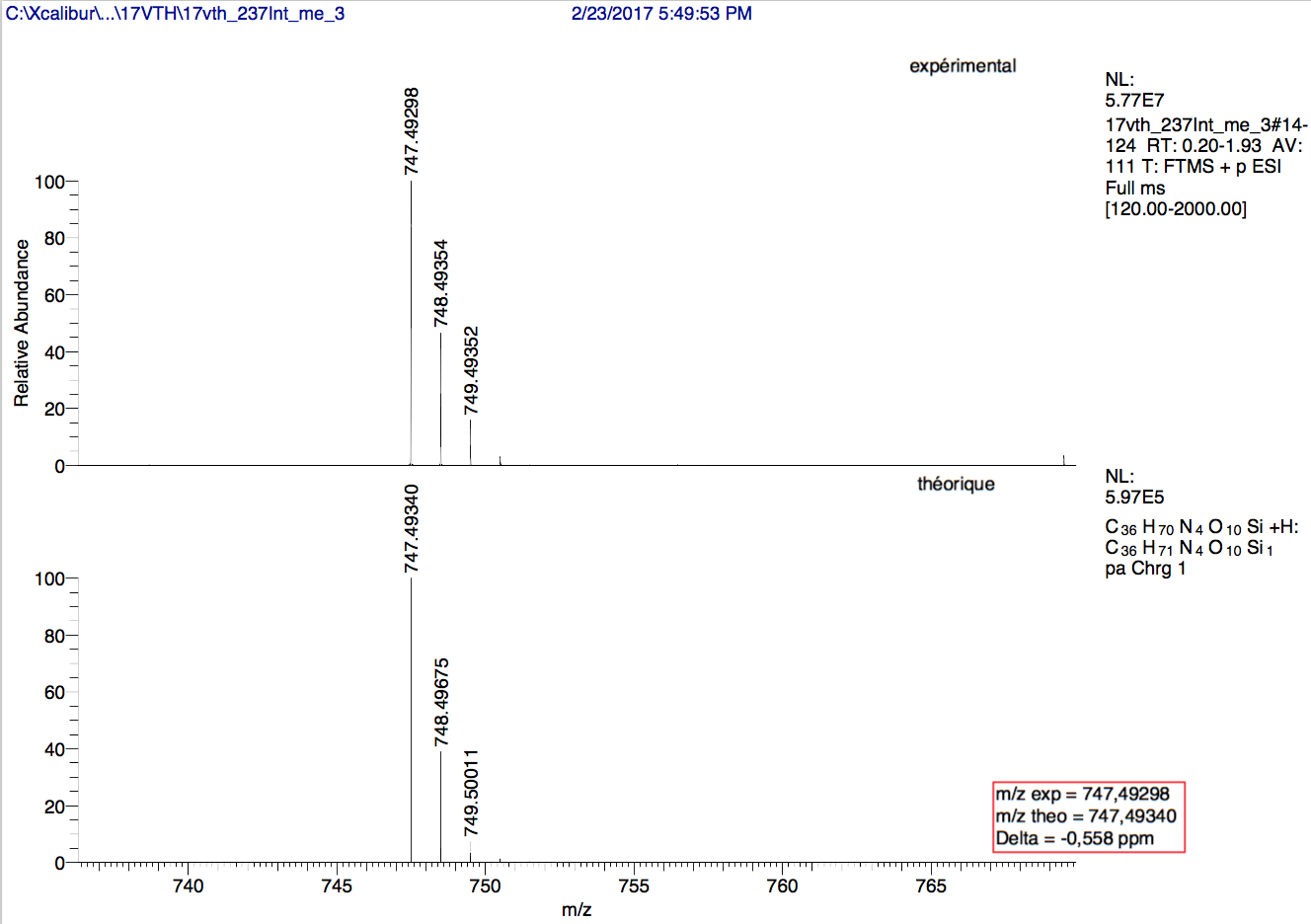


Figure S11. Mass spectrum of butyl protected APTES-NODAGA: A) experimental spectrum, B) simulated spectrum

A)

B)


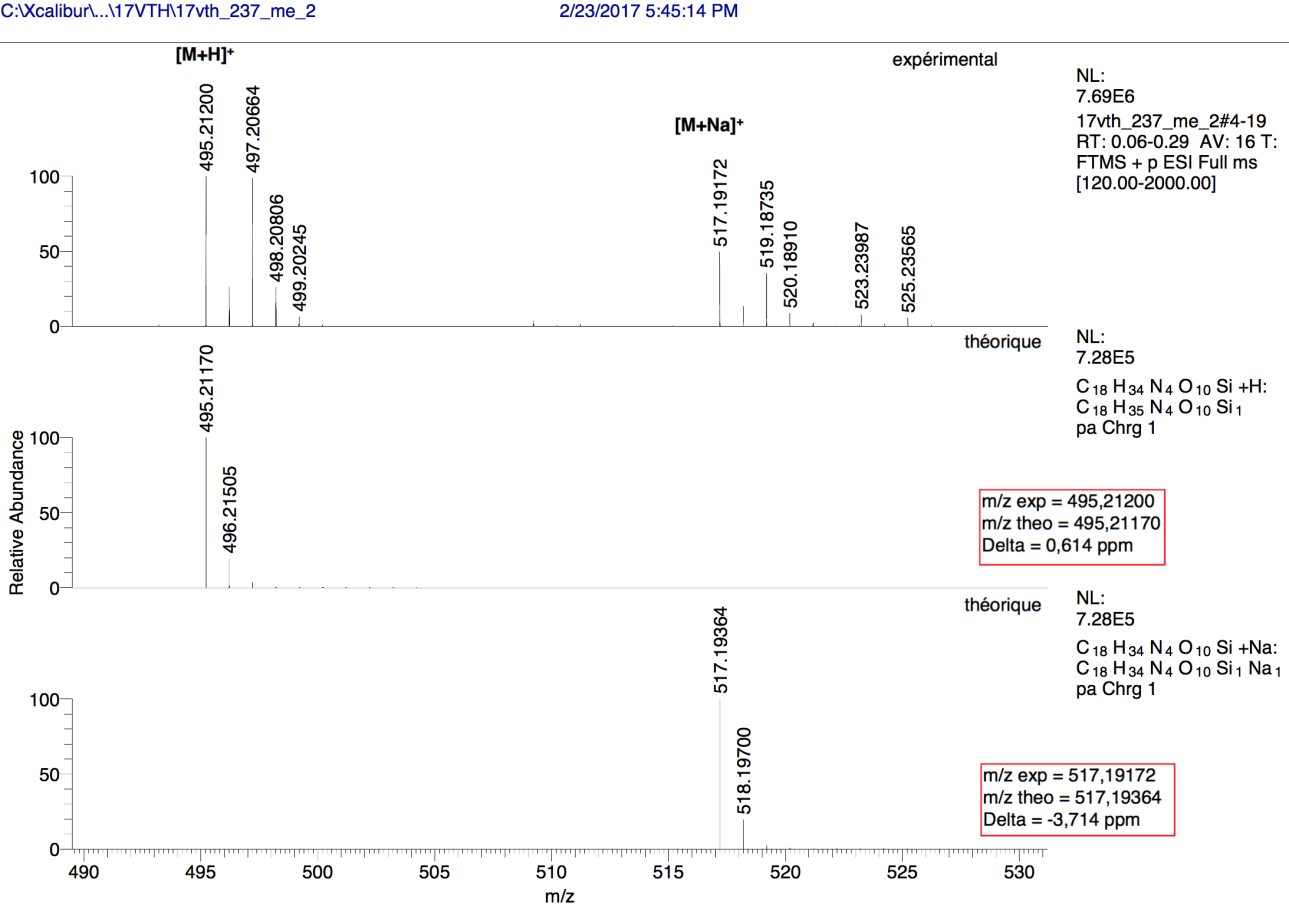


Figure S12. Mass spectrum of deprotected APTES-NODAGA: A) experimental spectrum, B) simulated spectrum

A)

B)

# References

(1) Plissonneau, M.; Pansieri, J.; Heinrich-Balard, L.; Morfin, J.-F.; Stransky-Heilkron, N.; Rivory, P.; Mowat, P.; Dumoulin, M.; Cohen, R.; Allémann, É.; et al. Gd-Nanoparticles Functionalization with Specific Peptides for ß-Amyloid Plaques Targeting. *J. Nanobiotechnology* **2016**, *14* (1).
